# Plasmid-Based Generation of Induced Neural Stem Cells from Adult Human Fibroblasts

**DOI:** 10.3389/fncel.2016.00245

**Published:** 2016-10-24

**Authors:** Philipp Capetian, Luis Azmitia, Martje G. Pauly, Victor Krajka, Felix Stengel, Eva-Maria Bernhardi, Mariana Klett, Britta Meier, Philip Seibler, Nancy Stanslowsky, Andreas Moser, Andreas Knopp, Gabriele Gillessen-Kaesbach, Guido Nikkhah, Florian Wegner, Máté Döbrössy, Christine Klein

**Affiliations:** ^1^Institute of Neurogenetics, University of LübeckLübeck, Germany; ^2^Department of Neurology, University of LübeckLübeck, Germany; ^3^Department of Neurosurgery, University of KielKiel, Germany; ^4^Laboratory of Stereotaxy and Interventional Neuroscience, Department of Stereotactic and Functional Neuroscience, University Medical Center FreiburgFreiburg im Breisgau, Germany; ^5^Department of Neurology, Hannover Medical SchoolHanover, Germany; ^6^Institute of Physiology, University of KielKiel, Germany; ^7^Institute of Human Genetics, University of LübeckLübeck, Germany; ^8^Department of Neurosurgery, University of Erlangen-NurembergErlangen, Germany

**Keywords:** direct reprogramming, plasmid based reprogramming, induced neural stem cells, episomal vectors, adult human fibroblasts, lineage conversion

## Abstract

Direct reprogramming from somatic to neural cell types has become an alternative to induced pluripotent stem cells. Most protocols employ viral expression systems, posing the risk of random genomic integration. Recent developments led to plasmid-based protocols, lowering this risk. However, these protocols either relied on continuous presence of a variety of small molecules or were only able to reprogram murine cells. We therefore established a reprogramming protocol based on vectors containing the Epstein-Barr virus (EBV)-derived oriP/EBNA1 as well as the defined expression factors Oct3/4, Sox2, Klf4, L-myc, Lin28, and a small hairpin directed against p53. We employed a defined neural medium in combination with the neurotrophins bFGF, EGF and FGF4 for cultivation without the addition of small molecules. After reprogramming, cells demonstrated a temporary increase in the expression of endogenous Oct3/4. We obtained induced neural stem cells (iNSC) 30 days after transfection. In contrast to previous results, plasmid vectors as well as a residual expression of reprogramming factors remained detectable in all cell lines. Cells showed a robust differentiation into neuronal (72%) and glial cells (9% astrocytes, 6% oligodendrocytes). Despite the temporary increase of pluripotency-associated Oct3/4 expression during reprogramming, we did not detect pluripotent stem cells or non-neural cells in culture (except occasional residual fibroblasts). Neurons showed electrical activity and functional glutamatergic synapses. Our results demonstrate that reprogramming adult human fibroblasts to iNSC by plasmid vectors and basic neural medium without small molecules is possible and feasible. However, a full set of pluripotency-associated transcription factors may indeed result in the acquisition of a transient (at least partial) pluripotent intermediate during reprogramming. In contrast to previous reports, the EBV-based plasmid system remained present and active inside the cells at all time points.

## Introduction

Reprogramming of somatic cells to induced pluripotent stem cells (iPSC) has opened up an entirely new perspective of obtaining patient-specific cell lines that can be further differentiated into the desired cell type (Takahashi et al., 2007). The employed culture techniques are, however, usually technically demanding and labor-intensive, the differentiation protocols complex and thus prompted the development of protocols for direct lineage conversion of somatic cells (Kelaini et al., 2014). In terms of direct neural reprogramming, two major approaches can be distinguished: The first one aims at reprogramming somatic cells into mature post-mitotic neurons (termed induced neurons (iN)), resulting in a limited number of derived neurons and requiring repeated reprogramming (Caiazzo et al., 2011; Pfisterer et al., 2011). The second approach aims at deriving neural precursors or stem cells that are still proliferative [induced neural stem cells (iNSC)] (Kim et al., 2011, 2014; Han et al., 2012), which increases the feasibility of applications demanding high cell numbers [for reviews see (Hermann and Storch, 2013) and (Mertens et al., 2016)]. The first protocols for reprogramming toward iNSC relied on retro- and lenti-viral expression systems (Kim et al., 2011; Thier et al., 2012). Concerns associated with the genomic integration of viral DNA led to protocols using either non-integrating Sendai virus (Lu et al., 2013) or episomal plasmids (Wang et al., 2012). However, these protocols were based on the addition of small molecules. A recent study was successful in overcoming this limitation by employing plasmid vectors with a set of reprogramming factors in conjunction with the oriP/EBNA1 expression system derived from the Ebstein-Barr virus (Kim et al., 2016). This expression system keeps plasmids as replicating episomes for a limited amount of time until being expelled from the cells at a later time point (Van Craenenbroeck et al., 2000). The published protocol demonstrated the feasibility of this approach for murine cells, however, without mentioning results for human cells. We therefore sought to establish an induction protocol for human adult fibroblasts to iNSC based on plasmid transfection of reprogramming factors in combination with basic neural medium and pro-neural factors without addition of small molecules. In addition to the characterization of proliferating and differentiated cells, we focused on the detection of persisting plasmid DNA and residual factor expression. Since the employed reprogramming factors are the same as in iPSC generation, it is perhaps not surprising that there is evidence from murine cells for a transient pluripotent step (Maza et al., 2015). We therefore tested for transient endogenous expression of the pluripotency-associated factor Oct3/4 as a potential indicator of such an occurrence in our human cellular model.

## Materials and Methods

### Cell Culture

We obtained fibroblast cultures from skin biopsies from three healthy human control individuals after giving informed consent (after approval of the ethics boards of the Universities of Freiburg and Lübeck). Cells proliferated in basic fibroblast medium (for exact medium composition see **Supplementary Table 1**). We repeated all reprogramming experiments at least three times for each line. Reprogramming started by a single round of electroporation of 500 K cells using the human dermal fibroblast kit together with a Nucleofector device (Lonza, Basel, Switzerland) following the manufacturer’s protocol (preset U-23). We used three plasmids in combination (1 μg of DNA of each plasmid): (1) pCXLE-hOct3/4-shp53-F (Addgene plasmid 27077) expressing the cDNA for human Oct3/4 under the control of a CAG promoter and a small hairpin RNA directed against human p53 under the control of the murine U6 promoter. (2) pCXLE-hSK (Addgene plasmid 27078) expressing the cDNA of human SOX2 and KLF4 separated by a 2A peptide under the control of a CAG promoter. (3) pCXLE-hUL (Addgene plasmid 27080; Okita et al., 2011) expressing the cDNA of human Lmyc and Lin28 separated by a 2A peptide under the control of a CAG promoter. All three plasmids contained the Epstein-Barr virus (EBV) latent origin of replication system (oriP) and expressed the Epstein-Barr virus nuclear antigen (EBNA1) under control of a CAG promoter. Control cells were transfected with 1 μg of a plasmid of a comparable size but with no reprogramming factor expression [pCXLE-eGFP (Addgene plasmid 27082; Okita et al., 2011)] expressing enhanced green fluorescent protein (eGFP) under control of a CAG promoter. Next, we plated the cells on a culture dish coated with BD Matrigel (MG, Cornig). Cells were visualized on an Axiovert 200M microscope (Zeiss, Jena, Germany).

Basic fibroblast medium was replaced step-wise every other day with N2B27 medium containing 20 ng/ml basic fibroblast growth factor (bFGF), 10 ng/ml FGF-4, and 10 ng/ml epidermal growth factor (EGF, all from Peprotech, Hamburg, Germany). Emerging neural structures were picked and replated on MG coated culture dishes in the same medium. When reaching confluence, cells were detached with accutase (life technologies, Carlsbad, CA, USA) and replated at a 1:5 ratio (referred to as *proliferative conditions* in the following text). For detailed analysis, a total of three fibroblast lines from healthy adult donors were reprogrammed with three clones per line.

For clonal analysis, cells were plated at very low density (50 cells/ml) on a culture dish coated with MG. Emerging single colonies were picked under the microscope and cultured accordingly. We used a total number of three clonal lines from three different fibroblast lines for further experiments. iPSCs serving as a control for expression of pluripotency-associated factors were generated by retroviral mediated overexpression of standard reprogramming factors (OCT4, SOX2, KLF4 and C-MYC) in human adult fibroblasts and cultured as described elsewhere (Seibler et al., 2011).

For differentiation and patterning experiments, cells were kept in N2B27 medium and bFGF, FGF4 and EGF were omitted. For differentiation, 20 ng/ml brain-derived neurotrophic factor (BDNF), 10 ng/ml glial cell line-derived neurotrophic factor (GDNF), 10 ng/ml insulin-like growth factor 1 (IGF-1, all from Peprotech, Hamburg, Germany), 0.5 mM dibutyril cyclic adenosine-monophosphate (dbcAMP, Enzo Life Sciences, Farmingdale, NY, USA), and 10 μM DAPT (Tocris, Ellisville, MO, USA) were added for 30 days.

### Karyotyping

Kryotyping was performed after passage 15 by Giemsa Trypsin banding. In short, colonies were incubated with a colcemid solution (10 μg/ml in HBSS) for 3 h to arrest cells in metaphase. Cells were treated with trypsin (0.25%) and the enzymatic reaction was stopped with Amniomax solution (Invitrogen, Darmstadt, Germany). Cells were centrifuged at 300 × *g* for 10 min and the pellet was resuspended in 4 ml hypotonic potassium chloride solution (5.62%). Cells were incubated for 5 min at 37°C and centrifuged at 300 × *g* for 10 min. Cells were resuspended and fixed in 5 ml glacial acetic acid and methanol (1:3) and subsequently centrifuged for 7 min at 350 × *g*. This step was repeated once. Finally, most of the supernatant was removed and cells were resuspended. Cell suspension was dropped onto cold slides and dried at 100°C for 1 h. Giemsa solution (5%) was added and incubated for 5 min. Slides were washed in distilled water two times, dried at room temperature and sealed with cover slips. Chromosomes of 10 – 12 cells in total were analyzed.

### Immunofluorescence and Confocal Imaging

Cells were plated on 12 mm glass coverslips (Karl Hecht, Sondheim, Germany) coated with poly-d-lysine (Sigma-Aldrich, St. Louis, MO, USA) and laminin (Roche, Basel, Switzerland). Cells were fixed with 4% PFA (Merck, Darmstadt, Germany), stained (for buffers, a detailed staining protocol and employed antibodies see **Supplementary Tables 2**–**4**) and mounted on glass slides (Menzel Gläser, Braunschweig, Germany). Imaging was performed with an LSM 710 confocal laser scanning microscope (Zeiss, Jena, Germany) equipped with the ZEN black software (Zeiss, Jena, Germany). For quantification, eight representative sample images were taken and cells counted with the ImageJ software.

### PCR Analysis

RNA or genomic DNA was extracted with the QIAamp mini DNA or RNA kit (Qiagen, Venlo, Netherlands) according to the manufacturer’s protocol. 500 ng of RNA were transcribed to cDNA with the First Strand cDNA Synthesis Kit (Thermo Fisher Scientific, Waltham, MA, USA). If possible, primers for cDNA analysis were designed to skip one or more introns; the sequences for plasmid-specific primers were adopted from the original publication (Okita et al., 2011; for a complete list of employed primers see **Supplementary Table 5**). qPCR reaction and analysis was carried out with the LC SYBR Green Mix (Fermentas, Vilnius, Lithuania) in a Lightcycler 480 (Roche, Basel, Switzerland, see **Supplementary Figure 1** for a detailed description of the conditions). Relative expression levels to the house keeping gene β-actin were calculated for cDNA and to GAPDH for genomic DNA by the relative standard curve method. (the latter values were divided by two in order to obtain values per genome). Standard curves were generated by serial dilutions of linearized plasmid templated containing the target sequence of the corresponding primer pair. Expression levels of endogenous Oct3/4 and SOX2 were evaluated by designing a primer pair with the reverse primer binding to the 3’-untranslated region of the SOX2-mRNA. A regular PCR was performed on cDNA or plasmid DNA with a taq polymerase (Thermo Fisher Scientific, Waltham, MA, USA, see **Supplementary Table 6** for a detailed description of the conditions) followed by gel electrophoresis (1% agarose). A 100-bp DNA ladder (New England Biolabs, Ipswich, MA, USA) was used to evaluate the correct product size.

### Electrophysiological Measurements

For electrophysiological measurements, cells were plated on coated glass coverslips and differentiated as described above; only the differentiation period was extended to 90 days. Patch pipettes were formed from borosilicate glass (Science Products, Hofheim, Germany) with a P1000 universal puller (Sutter Instruments, Novato, CA, USA) to final resistances of 3 to 4 MΩ when filled with the internal solution consisting of 153 mM KCl, 1 mM MgCl_2_, 10 mM HEPES, 5 mM EDTA and 2 mM Mg-ATP, adjusted to pH 7.3 with KOH (305 mOsm). The bath solution contained 142 mM NaCl, 8 mM KCl, 1 mM CaCl_2_, 6 mM MgCl2, 10 mM glucose and 10 mM HEPES, adjusted to pH 7.4 with NaOH (325 mOsm). Tetrodotoxin (TTX, 1 μM), tetraethylammoniumchloride (TEA, 10 mM), 2,3-dihydroxy-6-nitro-7-sulphamoyl-benzo(f)quinoxaline (NBQX, 10 μM), and memantine (100 μM, all purchased from Sigma-Aldrich) were diluted in the bath solution and applied via gravity using a modified SF-77B perfusion fast-step system (Warner Instruments, Hamden, CT, USA) as published (Wegner et al., 2008). Whole-cell patch clamp experiments were performed at 20–22°C under optical control (inverted microscope, Zeiss, Jena, Germany). Cells with leak currents <100 pA were used for further analysis. Whole-cell currents were low-pass filtered at 2.9 kHz, digitized at 10 kHz using an EPC-10 amplifier (HEKA, Lambrecht, Germany) and analyzed with Patch Master (HEKA).

### Statistical Analysis

Obtained results were computed with the help of a statistics software (GraphPadPrism 6.0, GraphPad Software, San Diego, CA, USA). For comparison of MAP2-positive cells between undifferentiated and differentiated states, we employed the paired *t*-test. The two-way ANOVA in combination with Dunnett’s multiple comparison test served for computing the time-dependent expression of endogenous Oct3/4. The effect of NBQX and memantine on neurons was calculated by a one-way ANOVA in combination with Bonferroni’s multiple comparison test. A result was considered significant at *p*-values < 0.05.

## Results

### Reprogramming and Establishment of iNSC Lines

**FIGURE 1:**
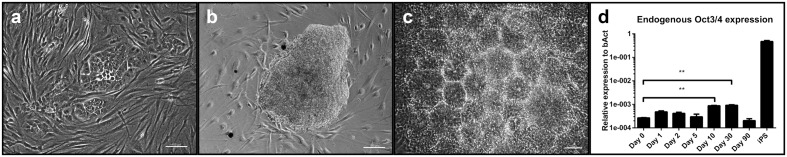
**Evaluation of the reprogramming process:** **(a)** Change in morphology with colony formation of transfected cells at day 7 post-transfection with the reprogramming plasmids. Scale bar = 100 μM. **(b)** A typical neural colony at the time of picking. Scale bar = 100 μm. **(c)** Rosette formation of cells after initial picking and replating. Scale bar = 50 μm. **(d)** Expression levels of endogenous Oct3/4 at different time points after transfection. Expression levels of iPSC are given as control. (three independent cell lines, two technical replicates, 2-way-ANOVA with Dunnett’s multiple comparison test, *p* = 0.0002, error bars correspond to standard deviation). ^∗∗^ = very significant.

After 10 days in culture, first morphological changes in cells transfected with the reprogramming plasmids became visible (**Figure 1a**). At about day 30 post-transfection, colonies reached a sufficient size (**Figure 1b**) for being picked manually and propagated under adherent conditions. Each round of transfection yielded between 10 and 20 expanding colonies, thus resulting in a reprogramming efficiency between 0.02 and 0.04% per cell (based on a number of 500,000 transfected cells). Mainly during the first passage, cells formed characteristic rosette-like structures (**Figure 1c**) and could be propagated for at least 25 passages without loss of proliferative capacity. Cells transfected with non-reprogramming plasmids did not show any morphological changes (**Supplementary Figure 4**).

### Transient Expression of Endogenous Oct3/4 during the Reprogramming Process

We checked for a potential intermediate (partial) pluripotent step during reprogramming by quantifying expression levels of endogenous Oct3/4 at different time points after transfection by qPCR(day 0 as baseline, day 1, 2, 5, 10, 30 and 90). Relative expression levels to the house-keeping gene beta-Actin (bAct) at day 10 and 30 showed a significant increase compared to baseline and decreased thereafter (**Figure 1d**, two-way ANOVA with Dunnett’s multiple comparison test, *p* = 0.0002). Relative expression levels of Oct3/4 in iPSC served as comparison (they were not included in the statistical analysis).

### iNSC under Proliferative Conditions

**FIGURE 2:**
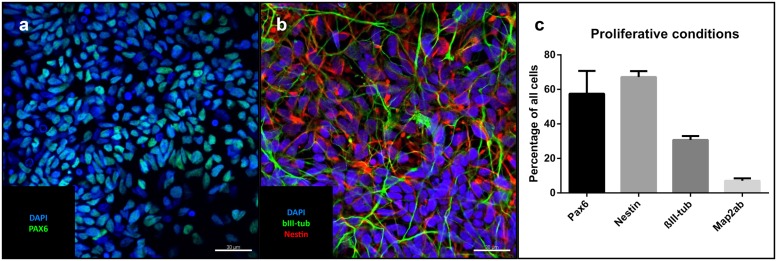
**Cells under proliferative conditions: Representative confocal images of iNSCs under proliferative conditions: **(a)** Pax6-positive nuclei (green), counterstaining with DAPI (blue).** Scale bar = 30 μm. **(b)** Nestin (red), bIII-tubulin (green), DAPI (blue). Scale bar = 30 μm. **(c)** Cell counts of different cell markers in relation to total cell number (means of eight high power fields from three iNSC cell lines, error bars correspond to standard deviation). Confocal images of cells after differentiation derived from a clonal line.

Under proliferative conditions (after 10 passages), 57% of the cells expressed the homeodomain protein PAX6, an early marker of neuroectodermal differentiation and radial glia of the dorsal telencephalon (Callaerts et al., 1997, p. 6). 67% were positive for the intermediate filament nestin expressed in neural stem cells, 30% stained positive for βIII-tubulin, a marker associated with neuronal progenitors and immature neurons (**Figures 2a–c**). The only non-neural cellular phenotype we sporadically identified consisted of alpha-smooth muscle actin-positive cells (**Supplementary Figure 2**). Pan-cytokeratin- and alpha-fetoprotein (AFP)-positive cells could not be identified (data not shown). Cells positive for the pluripotency marker NANOG were also absent (data not shown).

### Derivation of Clonal Lines and Analysis of Tripotency

**FIGURE 3:**
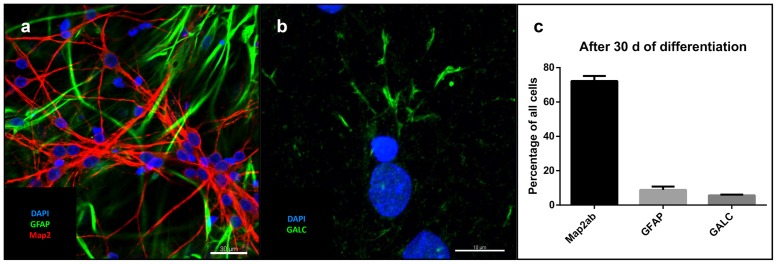
**Differentiation of iNSC:** **(a)** GFAP (green), MAP2 (red) and DAPI (blue). Scale bar = 30 μm. **(b)** GALC (green) and DAPI (blue). Scale bar = 10 μm. **(c)** Cell counts after differentiation of three clonal lines in relation to total cell number (means of eight high power fields from three iNSC cell lines, error bars correspond to standard deviation).

After removal of bFGF, FGF4, and EGF and addition of BDNF, GDNF, IGF-1, dbcAMP, and DAPT for at least 30 days, single cell-derived colonies on MG showed neural trilineage differentiation capacity, as evidenced by positive immunostaining for neuronal microtubule associated protein 2 (MAP2), astrocytic glial fibrillary acidic protein (GFAP; **Figure 3a**), and the oligodendrocyte marker galactosylceramidase (GALC; **Figure 3b**). Percentage of total cells (as determined by DAPI staining) was 72% for MAP2, 8.9% for GFAP and 5.7% for GALC (**Figure 3c**).

### Karyotyping, Evaluation of Residual Plasmid Expression, and Activation of SOX2 Expression

**FIGURE 4:**
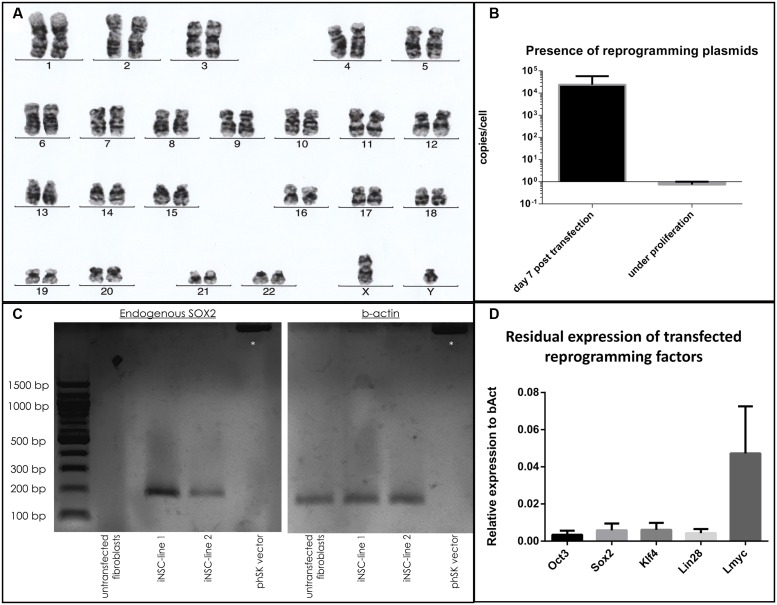
**Evaluation of genomic stability and continuous expression of reprogramming factors:** **(a)** Normal karyogram of an iNSC line. **(b)** Copy numbers of plasmid per genome as quantified by qPCR at day 5 after transfection and under proliferative conditions (two independent experiments with three cell lines, two technical replicates, error bars correspond to standard deviation). **(c)** Gel electrophoresis of a PCR with a primer pair specific for the endogenous SOX2 sequence (left): A reaction product is only present for the cDNA derived from iNSC lines, not from fibroblasts or when using the reprogramming plasmid carrying the SOX2 sequence. Equal levels of b-Actin for the cell lines are demonstrated on the right. Supercoiled plamid DNA is visible at the top (asterisk). **(d)** qPCR analysis of residual reprogramming factor expression in relation to the house keeping gene beta-actin (bAct) under proliferation of iNSCs (means of three independent iNSC lines, two technical replicates, error bars correspond to standard deviation).

Cells demonstrated a stable karyotype as determined by karyogram (**Figure 4a**). Although episomal vectors were used for reprogramming, cells contained 0.74 copies per cell under proliferative conditions, as determined by qPCR with a plasmid-specific primer pair, in contrast to 23879.4 copies per cell at day 5 post-transfection (**Figure 4b**). The absent peak in the melting curve for untransfected fibroblasts (**Supplementary Figure 3**) demonstrated the specificity of the assay. In iNSC lines, the presence of endogenous SOX2 mRNA could be demonstrated by PCR, while being absent in untransfected fibroblasts. Absence of a reaction product when using the reprogramming plasmid encompassing the SOX2 sequence as template demonstrated the specificity for endogenous SOX2 mRNA (**Figure 4c**). A variable but clearly measurable residual expression of reprogramming factors was detected by qPCR, in particular of *L-MYC* (**Figure 4d**).

### Differentiation and Neuronal Subtypes

**FIGURE 5:**
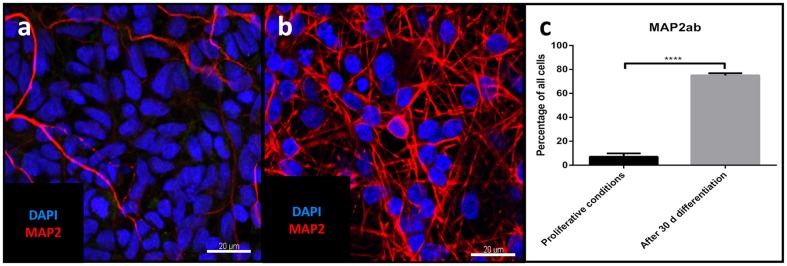
**Evaluation of neuronal maturation through differentiation:** Confocal image of MAP2-positive cells (red) before **(a)** and after differentiation **(b)**, nuclear counterstaining with DAPI (blue). Scale bar = 20 μm. **(c)** Change in percentage of MAP2-positive cells relative to total cells before and after differentiation (means of eight high power fields from three iNSC cell lines each, paired *t*-test, *p* < 0.0001). ^∗∗∗∗^ = extremely significant.

**FIGURE 6:**
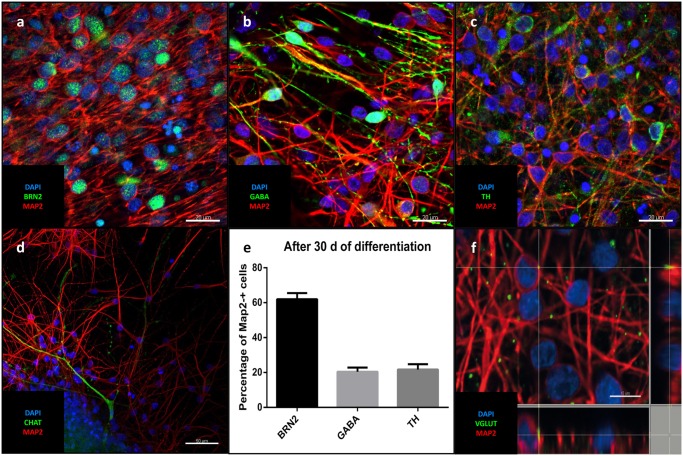
**Neuronal identity after differentiation: Representative confocal images of cells after 30 days of differentiation:** In green the upper-layer cortical marker Brn2 **(a)**, GABA **(b)**, TH **(c)**, CHAT **(d)**. **(a-d)** MAP2 (red), DAPI (blue). Scale bars = 20 μm **(a-c)**, 50 μm **(d)**. **(e)** Percentage of different neuronal markers in relation to MAP2-positive cells (means of eight high power fields from three iNSC cell lines, error bars correspond to standard deviation). **(f)** Puncate staining of VGLUT1 adjecent to neuronal soma and processes corresponding to glutamatergic synapses.

Differentiation for at least 30 days led to subsequent maturation of the iNSC represented by a significant increase (means of eight high power fields from four lines each, paired *t*-test, *p* < 0.001) of MAP2-positive cells with long and elaborate processes from 7% under proliferative conditions to 75% at the end of the differentiation (**Figures 5a–c**). Further quantification of the neuronal subtypes after 30 days of differentiation revealed 61.9% of the MAP2-positive cells to be BRN2-positive (a marker of glutamatergic upper-layer cortical neurons; **Figures 6a,e**). We tried staining glutamatergic neurons directly for vesicular glutamate transporter 1 (VGLUT1), however, the punctuate staining of synaptic terminals in the absence of a somatic signal prevented quantification (**Figure 6f**). 20.4% of differentiated neurons stained positive for GABA (**Figures 6b,e**), 21.7% were positive for tyrosine hydroxylase (TH, **Figures 6c,e**). Only very occasionally (<0.01%), neurons expressed choline-acetyltransferase (CHAT), the rate-limiting enzyme of acetylcholine synthesis. The latter exhibited a relatively large soma with very elaborate processes (however, void of MAP2, **Figure 6d**). Ctip2-positive (a marker for lower-layer cortical neurons) cells were absent (data not shown).

### Electrophysiological Assessment

**FIGURE 7:**
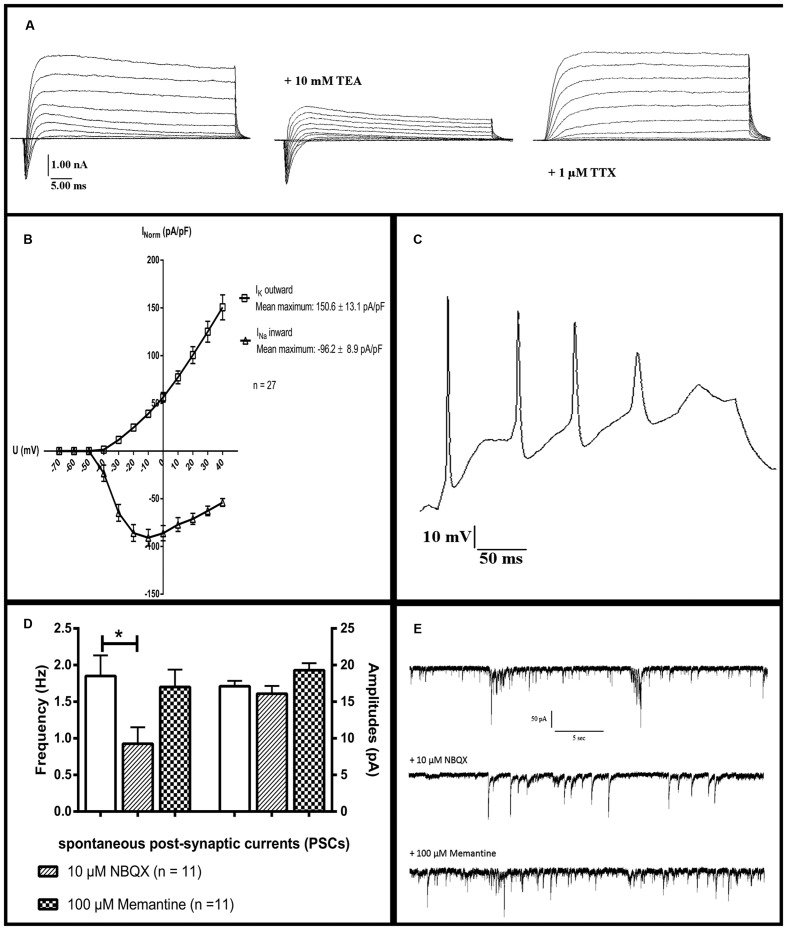
**Electrophysiological characterization: Whole-cell patch-clamp recordings of cells after 90 days of differentiation.** **(a)** The presence of sodium and potassium channels was demonstrated by short inward and long outward currents upon depolarization. Outward currents were blockable by the potassium channel inhibitor TEA, inward currents by the sodium channel blocker TTX. **(b)** Ion currents normalized for cell sizes based on the capacitance of the cell membrane (pA/pF). **(c)** A neuron firing multiple action potentials upon depolarization. **(d)** Significant reduction of the frequency but not amplitude of PSCs after application of the inhibitor for AMPA-receptors NBQX. The NMDA-receptor-antagonist memantine (100 μM) shows no significant effect on either parameter (11 neurons analyzed, one-way ANOVA, *p* = 0.028 for frequency after NBQX application, for the rest *p* > 0.05). **(e)** Representative tracings of PSCs of one neuron before and after the application of 10 μM NBQX or 100 μM memantine. TEA, tetraethylammoniumchloride; TTX, tetrodotoxin; NBQX, 2,3-dihydroxy-6-nitro-7-sulphamoyl-benzo(f)quinoxaline. ^∗^ = significant.

After 90 days of differentiation, neuronal properties of cells harboring a neuronal morphology were analyzed: Cells exerted a negative resting membrane potential of -37.5 ± 2.1 mV. Large outward currents were reliably induced by depolarizing voltage steps of 10 mV from a holding potential of -70 to 40 mV. These currents showed voltage dependence and kinetic characteristics of potassium currents, which were inhibited by the potassium channel blocker TEA (10 mM) applied to the extracellular solution (**Figure 7a**). In response to depolarization, cells also generated sodium inward currents that were blocked by the sodium channel blocker TTX (1 μM; **Figure 7a**). Peak currents were normalized for cell size based on the capacitance of the cell membrane (pA/pF, **Figure 7b**). In current-clamp experiments, 96% (*n* = 25/26) of the neurons fired TTX-sensitive APs with average amplitudes of 44.6 ± 2.3 mV and durations of 3.3 ± 0.3 ms, 24% (*n* = 6/26) fired multiple APs upon depolarization (**Figure 7c**). The functional properties of differentiated iNSC derived neurons are summarized in **Table 1**.

**Table 1:** Electrophysiological properties by whole-cell patch clamping of neurons after 90 days of differentiation (number of recorded neurons = 26, three independent cell lines).

Functional properties	
Peak Na +peak current	-96.2 ± 8.9 pA/pF
Peak K +peak current	150.6 ± 13.1 pA/pF
Resting membrane potential	-37.5 ± 2.1 mV
Membrane capacitance	23.2 ± 2.0 pF
Input resistance	608.4 ± 78.1 MOhm
Cells with APs	25 (96%)
Cells with multiple APs	6 (24%)
Cells with spontaneous APs	10 (40%)
AP amplitude	44.6 ± 2.3 mV
AP duration	3.3 ± 0.3 ms
AHP amplitude	19.6 ± 1.7 mV
Time to peak AHP	11.0 ± 0.8 ms


*AHP, after hyperpolarization; AP, action potential.*

Spontaneous post-synaptic currents (PSCs) were found in 40% of the cells (*n* = 10/26). Application of 10 μM of the potent and competitive AMPA receptor antagonist NBQX led to a significant reduction of the frequency but not of the amplitude of PSCs. Application of the non-competitive NMDA receptor antagonist memantine (100 μM) had no effect on either parameter (*n* = 11, one-way ANOVA, *p* = 0.028 for frequency after NBQX application, for the remainder *p* > 0.05, **Figures 7d,e**). Staining for vesicular glutamate transporter 1 (VGLUT1), demonstrated its presence on neuronal surfaces in culture after differentiation (**Figure 6f**).

## Discussion

Virus-mediated gene delivery requires higher biosafety levels and is more technically demanding than plasmid-based protocols. Therefore, plasmid-based transfection protocols allow the application of reprogramming in laboratories without a virus facility. For our protocol, we chose a well-established and effective reprogramming system (Okita et al., 2011). This system employs five transcription factors (Oct3/4, Sox2, Klf4, Lin28 and L-myc). A small hairpin RNA silencing p53 expression included for enhancing the reprogramming process, as it has been shown that p53 suppresses successful cellular reprogramming (Hong et al., 2009). While other protocols sought to reduce the number of factors employed (Mitchell et al., 2014), our priority was to increase the effectivity of the reprogramming process. Therefore, we accepted a higher number of reprogramming factors.

### Reprogramming and Establishment of iNSC Lines

Based on the published experience that employing basic neural medium in combination with growth factors (promoting growth of neural precursors and stem cells) was sufficient for reprogramming of murine fibroblasts (Kim et al., 2016), we avoided small molecules promoting neural induction (Lu et al., 2013). The rationale behind this approach was to interfere as little as possible with the ongoing reprogramming process, as many small molecules employed during neural reprogramming also serve as morphogens and could change regional identity (e.g., purmorphamine and CHIR99021; Kriks et al., 2011). The composition of the neural medium followed a protocol for reprogramming of murine fibroblasts (Kim et al., 2014), which includes the well-established growth factors FGF-2 and EGF (Hitoshi et al., 2004; Deleyrolle and Reynolds, 2009), but also the less commonly used factor FGF-4. Nonetheless, evidence exists for it promoting the proliferation of neural stem cells (Kosaka et al., 2006). We were able to show that transfected adult fibroblasts changed their morphology as early as 7 days after transfection, reaching an appropriate size for mechanical isolation after roughly 30 days. The efficiency of the method lies between 0.02 and 0.04%, i.e., the range reported in the original publication which used the plasmid constructs for reprogramming to iPSC (Okita et al., 2011). Another protocol overexpressing PAX6 and SOX2 by plasmid transfection in fibroblasts for reprogramming to neural precursor-like cells reported an efficiency of 0.05% (Maucksch et al., 2012). Protocols using retroviral particles achieved higher rates of reprogramming (0.5–1%; Ring et al., 2012). We would attribute this to the in general lower transfection efficiency of plasmid-based transfection protocols compared to retroviral infection. (For an overview of published integration-free conversion protocols to NSC see **Supplementary Table 7**).

### Transient Expression of Endogenous Oct3/4 during the Reprogramming Process

Combining the expression of pluripotency factors (Okita et al., 2011) with basic neural medium and neural growth factors (Ying et al., 2003; Hitoshi et al., 2004) in order to achieve neural reprogramming, raises the question whether cells transit through an intermediate pluripotent step. The first publication on iNSC addressed this issue by employing a pNANOG-eGFP construct as a marker of (temporary) pluripotency (Kim et al., 2011) and revealed that no NANOG-positive cells developed with the reported protocol for direct neural reprogramming. A recent publication, however, called these findings into question. This study employed an inducible polycistronic expression cassette of Oct3/4, SOX2, KLF4 and C-MYC in combination with a NANOG-CRE reporter system. The vast majority of murine fibroblasts temporally passed through a pluripotent step during transdifferentiation toward iNSC (Maza et al., 2015). As there is no published data for human cells, we addressed this in our work. As we found that human fibroblasts expressed low levels of transcription factors associated with pluripotency (e.g., Oct3/4), employing a reporter construct did not seem feasible. Therefore, we chose to quantify endogenous expression levels of Oct3/4 during the initial phase of reprogramming. Ten days after transfection, expression levels increased significantly and remained elevated until the first passage after 30 days. During long-term culturing, levels decreased again to the baseline level. Furthermore, upon long-term proliferation, we could not detect any NANOG-positive cells by immunofluorescence. Notably, we cannot determine whether the intermediate cell types possess bona-fide pluripotency. However, increased expression levels of Oct3/4 are associated with the epiblast stage *in vivo* and decrease in cells of the primitive neural plate (Iwafuchi-Doi et al., 2011, p. 5). However, the initial reports meticulously characterizing the expression of Oct3/4 during murine embryogenesis by *in situ* hybridization found this factor expressed in the primitive neuroectoderm (Palmieri et al., 1994, p. 3). Taken together, further studies will be necessary to conclusively answer the question of transient pluripotency during the reprogramming process. Obviously, employing a set of reprogramming factors unable to reprogram somatic cells to a pluripotent state might help avoiding this problem: Indeed, one recent study was successful in reprogramming somatic cells toward neural stem cells without overexpressing Oct3/4 (Kim et al., 2016).

### iNSC under Proliferative Conditions

After replating, cells homogeneously reformed into the characteristic rosette-like growth pattern known of neuroepithelial precursors *in vitro* (Elkabetz et al., 2008). As already reported for iPSC-derived culture systems, this cell type is not stable under cultivation under FGF/EGF-containing conditions and the proliferative NSCs acquire a phenotype reminiscent of radial glia (symmetric proliferation, bipolar morphology, Nestin and PAX6-positivity, and tripotency; Conti et al., 2005; Elkabetz et al., 2008; Conti and Cattaneo, 2010). As indeed the majority (67%) of the reprogrammed cells expressed nestin and 57% expressed PAX6 under proliferative conditions, they showed a corresponding morphology and proved able to derive neurons and glia in clonally derived cultures. Thus, there is a solid body of evidence indicating that they acquire features associated with NSC. However, 30% of cells stained positive for bIII-tubulin [considered the earliest marker for neuronal commitment (Fanarraga et al., 1999)], whereas MAP2 as a marker of mature neuronal cells was found in only 7% of the cells. This demonstrates that even under proliferative conditions, a certain degree of spontaneous neuronal commitment and even differentiation occurs, a feature also reported in the context of reprogramming protocols employing retroviral particles for gene delivery (Kim et al., 2011).

### Residual Expression of Reprogramming Factors

The mean number of persisting plasmid copies per cell was 0.7 (ranging between 0.0006 and 2.8 between lines). Our plasmids contained the EBV latent origin of replication system (oriP) and expressed the Epstein-Barr virus nuclear antigen (EBNA1). This allows replication during cell cycle while still persisting as episomal elements during long-term passaging with only very infrequent genomic integration (Westphal et al., 1998). As we did not perform fluorescence in-situ hybridization (FISH) on our cells, we cannot rule out a stable genomic integration. These findings differ from the original publication which employed the reprogramming plasmids to derive iPSCs and detected an ongoing presence of plasmid sequence only in two out of seven lines (Okita et al., 2011). Another study employing vectors containing the oriP/EBNA1 system for direct reprogramming could not detect ongoing expression of reprogramming factors (Kim et al., 2016). However, other studies evaluating the feasibility of oriP/EBNA1 as an expression system in human and murine stem cells demonstrated a prolonged expression (Ali Hosseini Rad et al., 2015).

We were able to demonstrate that reprogrammed cells expressed endogenous SOX2, a prerequisite for early neural precursor identity (Graham et al., 2003, p. 2; Ferri et al., 2013, p. 2). Conversely, fibroblast cultures did not express Sox2 before reprogramming. This argues in favor of the fact that expression of the reprogramming factors in conjunction with the neural medium led to a profound change in the cellular identity toward a neural stem cell lineage. However, due to the sustained expression of reprogramming factors, we cannot fully rule out the fact that iNSC identity would still be dependent on them.

### Differentiation and Evaluation of Neuronal Subtypes

As expected from neural stem cells, removal of mitogens, addition of neurotrophic factors (in our case BDNF, GDNF, IGF and dbcAMP) and the pro-neurogenic, NOTCH-inhibiting small molecule DAPT, led to differentiation, reflected by cells forming elaborate MAP2-positive processes but also an increase of the number of MAP2-positive cells. Astrocytes and oligodendrocytes were also present after differentiation of clonally derived iNSC, clearly demonstrating the tripotency of the proliferative cells. Further analysis of neuronal subtypes revealed a predominance of neurons with the upper-layer cortical marker BRN2 over GABA and TH and a virtual absence of CHAT-positive cells. It has been reported that even in the absence of morphogens, neural cells derived from iPSCs acquire a predominantly anterior identity (Pankratz et al., 2007). Ongoing culturing of neural stem cells depends on mitogens like FGF2 and maintains neurogenic potency, however, leads to a relaxation of the stringency of positional identities: NSCs derived from embryonic cortex ventralize partly in the presence of FGF2 during culturing and result in GABAergic neurons (Bithell et al., 2008). Based on our findings, iNSCs behave according to their counterparts derived from iPSCs or primary sources.

### Electrophysiological Assessment

After 90 days of differentiation, cells showed functional neuronal membrane properties including a negative membrane potential, fast inward sodium and outwardly rectifying potassium currents in 96% of analyzed cells. More mature functional properties like spontaneous firing of action potentials and the firing of multiple action potentials upon depolarization were only present in a fraction of cells (40 and 24%, respectively). Previously published reports on neurons from directly reprogrammed neural precursors did not provide a detailed analysis of electrophysiological properties. Our data is, however, in line with findings from iPSC-derived neurons, of which even after long-term differentiation, only a fraction of neuronal cells acquire a completely mature electrophysiological phenotype (Pre et al., 2014). Reasons for that are most likely suboptimal cellular culture conditions, as addition of glial cells to developing neurons enhances their maturity by secreted factors (Blondel et al., 2000; Boehler et al., 2007; Pre et al., 2014).

Based on the presence of the upper cortical layer marker BRN2 in 61.9% of MAP2-positive cells, we consider the majority of neurons to be glutamatergic. The spontaneous miniature synaptic activity was reduced by inhibition of AMPA but not of NMDA receptors, which is in line with the notion that maturation of glutamatergic synapses is associated with a shift from predominant NMDA to AMPA receptor types (Groc et al., 2006). Furthermore, all cells analyzed showed PSCs, further supporting maturity of glutamatergic synapses.

## Author Contributions

PC: Conceptual design, experimental procedures and data collection (reprogramming, culturing, qPCR, immunofluorescence), manuscript, funding. LA: Conceptual design, experimental procedures and data collection (reprogramming, culturing, qPCR, immunofluorescence), manuscript, funding. MP: experimental procedures and data collection (reprogramming, culturing, qPCR, immunofluorescence). VK: experimental procedures and data collection (culturing, qPCR). FS: experimental procedures and data collection (culturing, PCR). E-MB: experimental procedures and data collection (qPCR). MK: experimental procedures and data collection (reprogramming, culturing, qPCR, immunofluorescence). BM: experimental procedures and data collection (reprogramming, culturing, qPCR, immunofluorescence). PS: experimental procedure and data collection (iPS cells). NS: Conceptual design, experimental procedures and data collection (electroyphysiology), manuscript. AM: Conceptual design and manuscript. AK: Conceptual design, experimental procedures, and data collection (electroyphysiology). GG-K: Conceptual design, experimental procedures and data collection (karyotyping), manuscript, funding. GN: Conceptual design and funding. FW: Conceptual design, experimental procedures and data collection (electroyphysiology), manuscript, and funding. MD: Conceptual design and funding. CK: Conceptual design, manuscript, and funding.

## Conflict of Interest Statement

The authors declare that the research was conducted in the absence of any commercial or financial relationships that could be construed as a potential conflict of interest.
