# Supplementary material for: Plasmid-Based Generation of Induced Neural Stem Cells from Adult Human Fibroblasts
Source: Front Cell Neurosci. 2016 Oct 24;10:245. doi: 10.3389/fncel.2016.00245 (PMC5075569; doi:10.3389/fncel.2016.00245)
Supplement: Supplementary file 2 [file Data_Sheet_1.PDF]

Programs

Program Name	pre-incubation		
Cycles	1	Analysis Mode	None

Target (°C)	Acquisition Mode	Hold (hh:mm:ss)	Ramp Rate (°C/s)	Acquisitions (per °C)	Sec Target (°C)	Step size (°C)	Step Delay (cycles)
95	None	00:05:00	4.40		0	0	0

Program Name	amplification		
Cycles	45	Analysis Mode	Quantification

Target (°C)	Acquisition Mode	Hold (hh:mm:ss)	Ramp Rate (°C/s)	Acquisitions (per °C)	Sec Target (°C)	Step size (°C)	Step Delay (cycles)
95	None	00:00:10	4.40		0	0	0
55	None	00:00:10	2.20		0	0	0
72	Single	00:00:10	4.40		0	0	0

Program Name	melting curve		
Cycles	1	Analysis Mode	Melting Curves

Target (°C)	Acquisition Mode	Hold (hh:mm:ss)	Ramp Rate (°C/s)	Acquisitions (per °C)	Sec Target (°C)	Step size (°C)	Step Delay (cycles)
95	None	00:00:05	4.40		0	0	0
65	None	00:01:00	2.20		0	0	0
97	Continuous		0.11	5	0	0	0

Program Name	cooling		
Cycles	1	Analysis Mode	None

Target (°C)	Acquisition Mode	Hold (hh:mm:ss)	Ramp Rate (°C/s)	Acquisitions (per °C)	Sec Target (°C)	Step size (°C)	Step Delay (cycles)
40	None	00:00:30	2.20		0	0	0

Supplemental figure 1: Experimental conditions for qPCR.

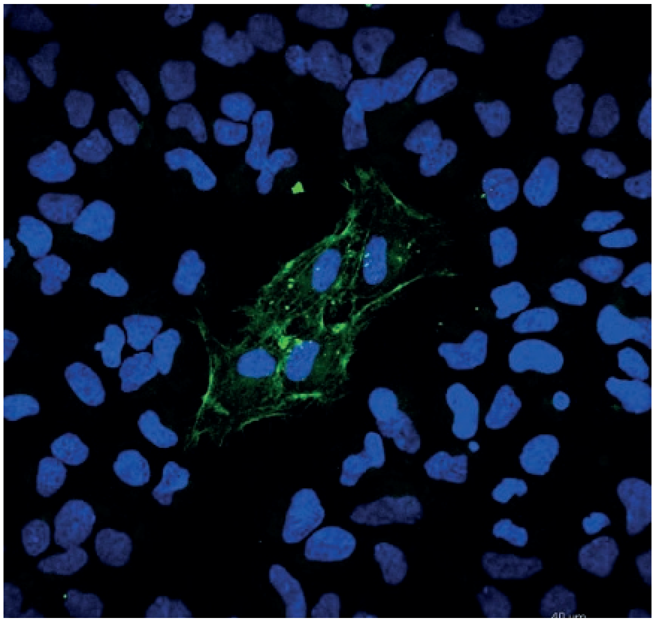

Supplemental figure 2: Only very rarely encountered SMA-positive mesodermal cell (green), counterstained with DAPI (blue). Scale bar = 40 µm.

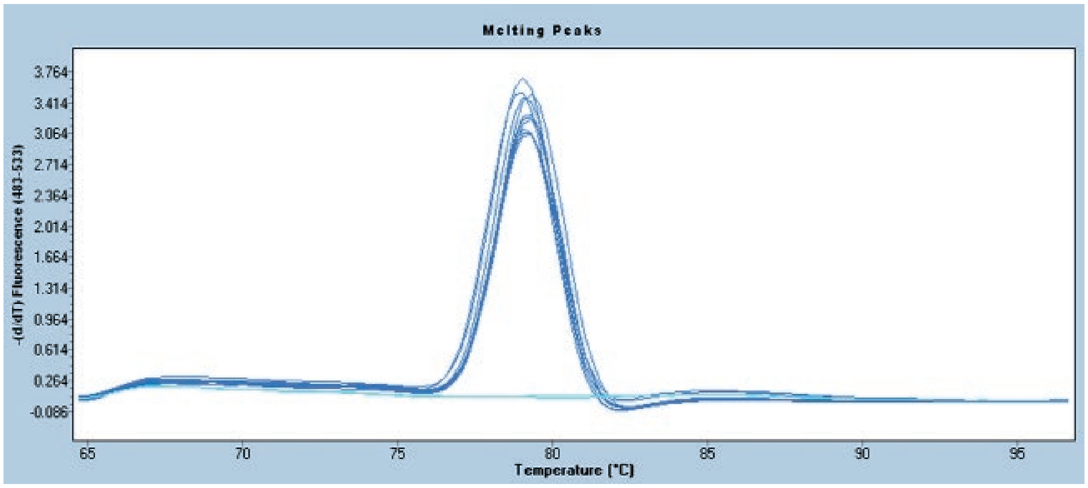

Supplemental figure 3: A peak in the melting curve demonstrates presence of plasmid DNA in transfected cells after qPCR with an EBNA1-specific primer pair (dark blue curves). Specificity is shown by absent peaks for DNA from untransfected fibroblasts (cyan lines).

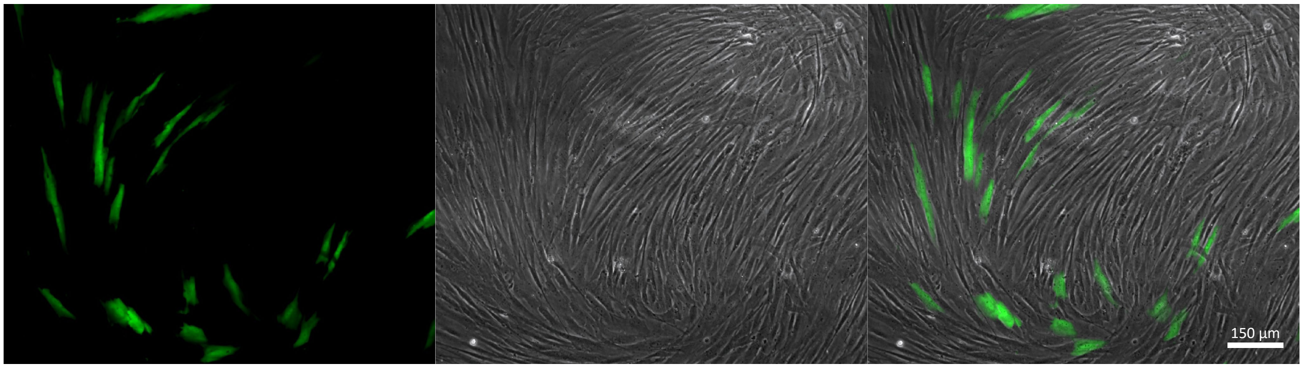

Supplemental figure 4: Fibroblasts transfected with the eGFP-expressing control plasmid (green) show no morphological changes in culture.
